# Surgical Treatment of Pectus Excavatum: The Boundary Between Pathologic and Aesthetic Need

**DOI:** 10.3390/jcm14010231

**Published:** 2025-01-03

**Authors:** Paola Ciriaco

**Affiliations:** Department of Thoracic Surgery, IRCCS San Raffaele Scientific Institute, 20132 Milan, Italy; ciriaco.paola@hsr.it

**Keywords:** pectus excavatum, Nuss procedure, vacuum bell, Ravitch procedure

## Abstract

**Background:** Pectus excavatum, also known as sunken chest or funnel chest, is a structural deformity of the anterior chest wall, characterized by an inward sternum. This condition can lead to respiratory and cardiovascular issues, although it is often addressed for aesthetic reasons. This perspective article reviews the experiences of multiple centers in treating pectus excavatum, to explore whether a clear boundary exists between pathological and aesthetic needs. **Methods:** The research was conducted on PubMed, using the following targeted search queries to identify relevant studies: “pectus excavatum and surgery”, and “pectus excavatum and conservative treatment.” Additional searches were performed for articles related to the psychological and emotional state of patients with pectus excavatum. **Results:** Over 2000 articles related to the topic were identified in the literature. Only primary studies from the past 20 years were included, with a focus on centers reporting a minimum of 30 to 50 cases annually. Nearly 60% of these centers perform the Nuss procedure, exclusively. Major complications occurred in 1–2% of cases, particularly when the procedure was performed without videothoracoscopy. Post-surgery, improvements in lung function and exercise capacity were reported, along with significant reductions in depression and anxiety. **Conclusions:** The decision to pursue surgical correction of pectus excavatum requires a thorough evaluation of both therapeutic and aesthetic factors. A patient-centered approach, considering both the physical and emotional aspects of the condition, is essential for achieving the best possible outcome.

## 1. Introduction

Pectus excavatum (PE), commonly referred to as sunken chest, is the most prevalent congenital chest deformity, affecting an estimated 1 in 300 to 400 live births [1]. It is characterized by a depression or concave appearance of the sternum (breastbone), causing the chest to appear inward rather than outward. This deformity primarily affects males, with a male-to-female ratio of approximately 3:1, indicating that the condition is more common in boys than in girls [1].

### 1.1. Clinical Presentation and Severity

While many cases are mild and may go unnoticed or remain asymptomatic, severe forms of pectus excavatum can have significant implications for both respiratory and cardiovascular health [2]. The inward displacement of the sternum can compress the heart and lungs, leading to restricted pulmonary function, decreased exercise tolerance, and in extreme cases, symptoms such as heart palpitations or arrhythmias, due to the alteration of the heart’s position within the chest [3,4]. In less severe cases, symptoms may be limited to cosmetic concerns, but in more pronounced deformities, physical limitations and discomfort can become problematic [5,6].

### 1.2. Genetic Associations

Pectus excavatum is most often an isolated congenital condition, meaning it occurs sporadically without an identifiable genetic cause. However, it can also be associated with certain genetic syndromes, including Marfan syndrome and Ehlers–Danlos syndrome [1,7]. Both syndromes involve connective tissue abnormalities that can affect multiple organ systems, including the skin, joints, and cardiovascular structures. In these cases, pectus excavatum may be one of several clinical manifestations, alongside other characteristic features of the syndromes, such as hyperflexible joints in Ehlers–Danlos syndrome or a tall, slender build in Marfan syndrome [7].

### 1.3. Diagnosis and Increasing Incidence

The diagnosis of pectus excavatum is typically made through clinical examination, supported by imaging studies such as chest X-rays, CT scans, or MRI, to assess the degree of deformity and any associated complications [8,9]. There has been an observed increase in the diagnosis of pectus excavatum in recent years. This trend is partly attributed to an increased awareness of the condition, advancements in imaging technology, and a broader understanding of the long-term health consequences of untreated deformities [10]. Additionally, many individuals may seek medical advice for pectus excavatum during adolescence or young adulthood, due to the social and psychological impact of the condition [5,10].

### 1.4. Non-Invasive Treatment Approaches

The treatment of pectus excavatum depends largely on the severity of the deformity, the presence of symptoms, and the patient’s age [8,9]. For many individuals, particularly those with mild cases, observation and conservative management may be sufficient. In these instances, regular monitoring is employed to track any changes over time, while physical therapy is often recommended to improve posture and strengthen the chest muscles, which may help minimize functional limitations [11].

For children and adolescents with mild to moderate pectus excavatum who still have growing chest walls, the vacuum bell is an increasingly popular non-invasive treatment option. The vacuum bell is a device that creates negative pressure around the chest, with the goal of gradually reshaping the sternum and improving the chest wall’s appearance. It is typically used for patients who have not yet reached full skeletal maturity, as the chest wall is more responsive to external forces during the growth period. Over time, consistent use of the vacuum bell can help elevate the depressed sternum, leading to improvements in both aesthetic appearance and respiratory function [12].

Some authors propose the use of a brace, which is generally prescribed for pectus carinatum. This device applies compression below the depressed area of the sternum. Its association with specific physiotherapy is reported to yield satisfactory results [11].

### 1.5. Surgical Interventions

For more severe forms of pectus excavatum, or in cases where non-invasive methods have been ineffective, surgical intervention is often required. There are two primary surgical techniques employed for the correction of pectus excavatum: the Nuss procedure and the Ravitch procedure [13,14]. The Nuss procedure is a minimally invasive surgery, which is commonly performed on adolescents and young adults with moderate-to-severe deformities. The procedure involves the insertion of a curved metal bar beneath the sternum, which is then used to elevate the depressed chest wall. The bar is typically left in place for two to three years to allow the chest to maintain its new shape while the bones and cartilage remodel. The procedure has a relatively short recovery time, and it is associated with less scarring compared to traditional open surgeries. The Ravitch procedure, in contrast, is a more invasive, open-chest surgery that involves the removal of abnormal cartilage and the repositioning of the sternum. This technique is often chosen for more complex or severe cases of pectus excavatum, or when the Nuss procedure is not suitable due to the patient’s specific anatomy. While the Ravitch procedure has a longer recovery time and involves larger incisions, it is considered a reliable option for correcting significant chest wall deformities, and can provide excellent long-term outcomes [14]. The purpose of this article is to analyze the experiences of major centers worldwide that are involved in the surgical treatment of pectus excavatum, aiming to delineate the boundary between therapeutic needs and purely aesthetic concerns for patients affected by this thoracic deformity [15].

## 2. Materials and Methods

An electronic search was conducted in the PubMed database in November 2024, for each of the following domains: type of surgical approach, namely Ravitch procedure and Nuss procedure; operative and perioperative complications; conservative treatment; and emotional and psychological well-being of patients. The specific search terms used were “pectus excavatum and surgery” and “pectus excavatum and conservative treatment”, “pectus excavatum and mental health”. Key words were: “pectus excavatum”, “Nuss procedure”, “Vacuum Bell” and “Ravitch procedure”. The titles and abstracts of all articles were checked for eligibility. The search was limited to the past 20 years, with a particular focus on centers handling a caseload of at least 30–50 patients per year [15]. Case reports, case series, and papers discussing also other chest wall deformities were excluded from the review (Table 1).

## 3. Results

More than 2000 related papers were found. After the screening, 26 papers in total were included in this review. Most papers focused on data related to the Nuss procedure, while a few analyzed data regarding the Ravitch technique [14,15,16,17]. Clinically significant perioperative complications were reported for around 7.7% for the entire population, with the incidence decreasing in centers with the highest annual case volume [15]. The optimal annual case volume was determined at 73 cases per year [15]. The meta-analysis published by Daemen, on 13,352 patients enrolled across 49 centers, reports that the most common minor complication was poor healing of the surgical incision site, which occurred in 3.5% to over 70% of cases [15]. Pleural effusion and pneumothorax followed, with an incidence of 2% [16]. Major complications included cardiopulmonary injuries, which occur in approximately 1–2%, especially in Nuss procedures performed without the assistance of video-assisted thoracoscopic surgery [16,17,18]. Focusing on the number of corrective bars used, positioning of two bars during the Nuss procedure is associated with a higher rate of postoperative complications [19]. The total number of complications, hematoma, the need of adjunctive chest tube, and bar displacement reach a statistical significance between the two groups (*p* < 0.001) [18,19]. The study by Huerta and colleagues reports a total of 10,053 patients with surgically treated pectus excavatum, with 86% (*n* = 8673) receiving the Nuss procedure, and 14% (*n* = 1380) undergoing the Ravitch procedure [14]. Among the reporting hospitals, 60% exclusively performed the Nuss procedure. A comparison between the Ravitch procedure and the Nuss procedure shows that the former carries higher risks of the pneumothorax requiring drainage (5.1% vs. 2.2%), bleeding (2.4% vs. 0.6%), prolonged air leaks (0.9% vs. 0.3%), and respiratory failure (1% vs. 0.3%), as well as longer hospital stays (4 vs. 3 days) (*p* < 0.05). Furthermore, Ravitch repairs were associated with higher total hospital costs (USD 18,670 vs. USD 17,462, *p* < 0.001) [13,14,17].

The Nuss procedure being performed in adults has been described as having complications, including steel bar displacement in 4.5% of patients, surgical site infection in 2.9%, pneumothorax in 2.7%, pleural effusion in 2.5%, and chronic pain in 2.2% of patients. Reoperation was necessary in 2.4% of cases for various reasons, including bleeding, the removal of the bar due to persistent pain, and bar displacement. Bar displacement is reported as a complication in 4.5% of cases [17,20].

Functional limitations in physical activity have been well documented in patients with pectus excavatum. Filaire and colleagues conducted an in-depth analysis of the hemodynamic changes that occur during exercise in patients with pectus excavatum, highlighting the physiological challenges that these individuals face during physical activity [21]. Their study demonstrated that patients with this condition experience significant reductions in key exercise parameters, including their peak power output, peak oxygen consumption (VO_2_), and peak cardiac output, compared to healthy controls.

Specifically, the study found that the peak power output of patients with pectus excavatum was significantly lower, with an average of 178 ± 9 W, compared to 286 ± 11 W in the control group. This decrease in power output likely reflects their reduced cardiovascular and muscular efficiency during physical exertion. Similarly, peak VO_2_, which is a critical measure of aerobic capacity, was also substantially lower in the patient group (35.6 ± 1.2 mL O_2_·kg^−1^·min^−1^ vs. 52.1 ± 1.5 mL O_2_·kg^−1^·min^−1^), indicating impaired oxygen utilization during exercise. Furthermore, peak cardiac output, a marker of the heart’s ability to pump blood effectively during exercise, was also reduced in patients with pectus excavatum (14.6 ± 0.6 L/min vs. 22 ± 1.6 L/min), further underscoring the cardiovascular limitations these individuals face.

However, improvements in lung function and exercise capacity have been reported in patients undergoing the Nuss procedure. Studies have shown that lung function, as measured by spirometry parameters such as forced vital capacity (FVC), forced expiratory volume in one second (FEV_1_), forced expiratory flow at 25% of FVC (FEF_25_), and the FEV_1_/VC ratio, improves significantly after surgery. These improvements are likely a result of correcting chest wall deformity, which reduces the mechanical constraints on the lungs, and increases thoracic volume. The postoperative values for these parameters are consistently higher than preoperative levels, reflecting enhanced pulmonary function and improved capacity for physical exertion [22].

In addition, several studies have analyzed the relationship between pectus deformity, as measured by the Haller index and correction index, and cardiopulmonary limitations. Some authors have reported that a Haller index greater than 3.6 is associated with pulmonary dysfunction, but not with cardiac dysfunction [23]. Other studies have found that resting pulmonary function test patterns show a poor correlation with the anatomical extent of the pectus defect, and lung volumes during exercise are only weakly associated with the correction index [24].

Studies analyzing the emotional well-being of patients with pectus excavatum have identified two categories: those who believe that chest deformity affects their life, and those who do not perceive it as impactful. Adolescents represent the age group in which emotional fragility is most pronounced [5]. The study by Luo L and colleagues describes the influence of the Nuss surgical procedure on mental health in Chinese patients and identify the predictors of psychological status for pectus excavatum [10]. They reported that some of the parameters, such as somatization, interpersonal sensitivity, depression, and anxiety after surgery, were significantly better than those before surgery (*p* < 0.05). On the other hand, the obsessive-compulsive symptoms, hostility, terror, paranoia ideation and psychoticism did not improve after surgery (*p* > 0.05) [10].

## 4. Discussion

The decision to pursue the surgical correction of pectus excavatum, commonly referred to as sunken chest or funnel chest, is multifaceted, and requires careful, individualized evaluation. This evaluation encompasses both therapeutic-medical and aesthetic-psychological factors, each of which plays a critical role in the overall treatment plan [8,9]. The evaluation to be made is related to the risk/benefit ratio of performing surgery for pectus excavatum solely to address aesthetic concerns.

While pectus excavatum is often recognized for its physical manifestations, one of the most profound impacts of deformity is its psychological toll. For some individuals, even mild to moderate forms of pectus excavatum can result in significant emotional distress. The sunken chest may become a source of self-consciousness, leading to social anxiety, diminished self-esteem, and a negative body image. This can have profound social and emotional consequences, particularly during adolescence, a period marked by heightened sensitivity to physical appearance [10].

For these individuals, cosmetic surgery may be pursued, not because of functional impairment, but because the deformity hinders their sense of self-confidence and social integration. Even with the absence of respiratory or cardiovascular issues, the psychological relief from a corrected chest wall shape can significantly enhance overall well-being. In some cases, the aesthetic improvement following surgery can lead to better mental health outcomes, restoring a sense of normalcy and enhancing quality of life [5].

The decision to pursue surgical treatment should be based on a comprehensive evaluation of both the physical and psychological aspects of the condition. Surgeons must engage in open, empathetic conversations with patients to fully understand their motivations, expectations, and concerns. A patient-centered approach that considers the individual’s quality of life, functional limitations, and aesthetic desires is essential in making an informed decision regarding surgical intervention. This approach can help clarify the rationale for surgery, ensuring that the treatment plan aligns with the patient’s overall well-being and personal goals [8,9,10].

This perspective underscores the importance of addressing psychological needs alongside physical symptoms when considering surgical intervention. Many patients, especially those with mild or moderate forms of deformity, may choose surgery to regain confidence and avoid the stigma that can sometimes accompanies visible physical differences [10]. It is important for healthcare providers to recognize that cosmetic surgery for pectus excavatum, though not medically required, can still be a valid and crucial treatment option for individuals suffering from emotional and psychological burdens related to their appearance.

On the other hand, for individuals with more severe forms of pectus excavatum, the decision to undergo surgery is often driven by functional impairments that affect respiratory and cardiovascular function. In these cases, deformity is not just a cosmetic issue, but a functional one that directly impacts daily life [11,14,21].

Severe pectus excavatum can compress the lungs, limiting their ability to expand fully. This can lead to reduced lung capacity, exercise intolerance, shortness of breath, and fatigue. These symptoms may become more pronounced during physical exertion, or even during rest in extreme cases [22]. In severe cases, the inward displacement of the sternum can exert pressure on the heart, leading to possible arrhythmias, reduced cardiac output, and other cardiovascular symptoms. As a result, individuals may experience palpitations, chest pain, or diminished cardiovascular fitness [21]. Analysis about the relationship between pectus deformity, as measured by the Haller index and correction index, and cardiopulmonary limitations have been reported [23,24]. The Haller index, a commonly used radiologic measure of pectus excavatum severity, reflects the ratio of the transverse diameter of the chest to the anteroposterior diameter at the level of the diaphragm. A higher Haller index typically indicates a more severe deformity. Some authors have reported that a Haller index greater than 3.6 is associated with pulmonary dysfunction, but not with cardiac dysfunction [23]. This suggests that, as the severity of the chest wall deformity increases, there may be a progressive impairment of lung function, while the heart’s ability to pump blood remains relatively unaffected, at least in the early stages.

Interestingly, despite these associations, several studies have shown that resting pulmonary function test patterns do not strongly correlate with the anatomic extent of the pectus defect. In fact, the degree of deformity, as quantified by the Haller index or the correction index, has been found to have a limited predictive value for baseline lung function. This observation suggests that the relationship between the chest wall deformity and pulmonary function is more complex than previously thought, and other factors, such as muscle strength, postural changes, and compensatory mechanisms may play significant roles. Moreover, lung volumes during exercise, specifically measures of dynamic lung function, have been shown to be only weakly correlated with the correction index. This weak association indicates that, while severe pectus excavatum may affect lung expansion and function at rest, the impact on exercise-induced respiratory mechanics may be less pronounced, possibly due to adaptive mechanisms that kick in during physical activity [23,24].

These findings highlight the need for a comprehensive assessment of both static and dynamic pulmonary function in patients with pectus excavatum. Furthermore, they underscore the importance of evaluating not only the structural characteristics of deformity, but also the functional consequences it has on cardiopulmonary health, particularly in relation to exercise capacity. While the Haller index remains a valuable tool for assessing the severity of the condition, its ability to predict functional impairment may be limited, suggesting that a multidimensional approach is necessary to fully understand the impact of pectus excavatum on respiratory and cardiovascular performance.

For patients experiencing these symptoms, surgery is viewed primarily as a therapeutic intervention to restore normal physiological function. Correcting the chest wall and alleviating the pressure on the heart and lungs can result in significant improvements in respiratory function, exercise capacity, and overall health. In these situations, the decision to pursue surgery is generally not questioned, as deformity is seen as having a clear, negative impact on health [8,9,13,14].

Corrective surgery for pectus excavatum, particularly the Nuss procedure, is potentially associated with major complications that may, in part, be due to the learning curve and, consequently, avoidable. The learning curve is facilitated by the higher number of cases performed annually, and centers with an annual caseload of around 70 patients report a lower incidence of complications and a shorter postoperative hospital stay [15]. Daemen reports an overall complication rate of 7.7%, but this incidence, when analyzed based on annual case volume, varies from 5.1% in centers performing the highest number of cases to 14.7% in those with fewer than 30 patients per year [15]. Another factor that differs significantly with respect to activity volume is the postoperative hospital stay, which is significantly shorter in centers where more surgeries are performed. This is not only due to improved surgical performance, but also to advancements in postoperative pain management strategies, which go hand in hand with the surgical learning curve [25,26].

The key to effective treatment for pectus excavatum lies in a balanced, patient-centered approach, which acknowledges and addresses both the physical and emotional aspects of the condition. This holistic approach requires healthcare providers to consider not only the degree of physical deformity but also the psychological and social implications of the condition for the patient [5,6,7,8,9,10].

To ensure the best possible outcome, a multidisciplinary team is often involved in the decision-making process. This team may include:

- Surgeons specializing in pectus excavatum correction (e.g., pediatric surgeons or thoracic surgeons) who are responsible for assessing the medical need for surgery.
- Psychologists or counselors evaluating the emotional impact of deformity, particularly for adolescents or young adults who may be facing significant body image issues.
- Pulmonologists assessing lung function and determine whether respiratory symptoms are clinically significant.
- Cardiologists evaluating the impact of the deformity on heart function, especially in more severe cases.
- Physical therapists to help assess and manage any limitations in physical activity that could be exacerbated by the deformity [25].

By considering both objective clinical data (such as lung and heart function) and subjective emotional responses (such as body image and self-esteem), healthcare providers can develop a treatment plan that best meets the individual needs of the patient [10].

The choice of surgical procedure, whether the Nuss procedure, Ravitch procedure, or another less invasive approach, depends on the patient’s specific condition, including the degree of deformity, age, overall health, and personal goals [13,14].

Each of these approaches can have distinct benefits depending on whether the focus is on improving functional capacity or on aesthetic enhancement. However, regardless of the surgical technique used, the goal remains the same: to improve quality of life by addressing both physical and psychological factors.

Long-term outcomes following surgical treatment for pectus excavatum underscore the importance of addressing both functional and aesthetic concerns. Numerous studies have demonstrated high levels of patient satisfaction post-surgery, particularly regarding improvements in self-image, physical function, and overall quality of life. However, it is crucial to continue monitoring patients for potential complications, such as recurrence of the deformity or issues related to the surgical repair, and to provide ongoing support, especially for younger patients who may be navigating body image issues during critical developmental stages [8,10,12].

## 5. Conclusions

The decision to pursue surgical correction of pectus excavatum involves a careful evaluation of both therapeutic and aesthetic considerations. For some individuals, deformity may cause significant psychological distress, leading to social and emotional consequences, even in the absence of substantial functional impairment. In these cases, surgery may be pursued more for cosmetic reasons than for direct medical necessity. For other patients, particularly those with more severe forms of pectus excavatum, the decision to undergo surgery is often driven by functional concerns, such as respiratory or cardiovascular symptoms, which significantly impact their quality of life. In these instances, surgical correction is typically viewed as a therapeutic intervention to restore normal physiological function, rather than a purely aesthetic procedure.

Future studies should focus on the actual need for surgical correction of pectus excavatum in cases where psychological factors are the predominant motivation.

The literature shows that surgical intervention for the correction of pectus excavatum can be associated with significant major complications. It is important to determine whether the sole psychological and aesthetic motivation, reported as a distress that limits normal social life, can justify the surgical indication.

This article has the limitation of not being a systematic review of the literature, nor a meta-analysis. The research may have also been limited by being the work of a single person. A prospective analysis comparing conservative treatment versus surgical treatment of pectus excavatum could provide more details on the surgical indication.

## Figures and Tables

**Table 1:** Search strategy.

Items	Specification
Date of search	November 2024
Sources searched	PubMed
Domains	Type of surgical approach, operative and perioperative complications, conservative treatment, emotional and psychological well-being
Key words	Pectus excavatum, Nuss Procedure, Vacuum Bell, Ravitch procedure
Search terms used	Pectus excavatum and surgery; pectus excavatum and conservative treatment; pectus excavatum and mental health
Time frame	Last 20 years
Inclusion criteria	Centers with a caseload of at least 30–50 patients per year
Exclusion criteria	Case reports, case series, papers discussing also other chest wall deformities
